# An Interesting Case of Refractory Kawasaki Disease With Co-infection

**DOI:** 10.7759/cureus.61726

**Published:** 2024-06-05

**Authors:** Ratan Kumar, Adyasha Mishra, Kumar Diwakar, Sanjay K Tanti

**Affiliations:** 1 Pediatric Intensive Care Unit, Tata Main Hospital, Jamshedpur , IND; 2 Pediatrics, Tata Main Hospital, Jamshedpur, IND; 3 Pediatrics, Manipal Tata Medical College, Manipal Academy of Higher Education (MAHE) Tata Main Hospital, Jamshedpur, IND

**Keywords:** uti, co-infection, immunoglobulin, refractory kawasaki disease, kawasaki disease, ivig

## Abstract

Kawasaki disease (KD), formerly called mucocutaneous lymph node syndrome, is one of the common vasculitides of childhood. KD most commonly occurs in children over six months up to five years of age, although it can occur in young infants, older children, and adults. Early diagnosis is critical to achieving optimal treatment. We present a case of a three-year-old female child who was admitted with a fever for five days and fulfilled the diagnostic clinical criteria for KD. She was given intravenous immunoglobulin (IVIG) and aspirin. However, the fever persisted, and a urine culture showed the growth of Klebsiella pneumoniae. We started an antibiotic based on her sensitivity. Since fever spikes were not subsiding, she was given a repeat dose of IVIG along with an oral corticosteroid for refractory KD, after which she showed clinical improvement. This case highlighted that refractory KD can coexist with infection.

## Introduction

Kawasaki disease (KD) is a systemic inflammatory disorder manifesting as vasculitis with a predilection for the coronary arteries [1-3]. It is typically a self-limited condition in which fever and manifestations of acute inflammation usually last for an average of 12 days without therapy [3]. However, complications such as coronary artery aneurysms, decreased myocardial contractility leading to heart failure, myocardial infarction, arrhythmias, and peripheral arterial occlusion may develop and lead to significant morbidity and mortality. The etiology of KD remains unknown [4]. Infection by one or more agents in genetically predisposed children may result in KD [5]. Some of these common bacterial or viral pathogens are parvovirus B19 [6], Propionibacterium [7], and human bocavirus [8]. The outcome of KD is mostly favorable when treated timely with intravenous immunoglobulin (IVIG). Refractory KD is a condition in which fever persists despite an initial dose of IVIG. Co-infection is not very uncommon with KD. However, co-infection in a case of IVIG-resistant KD causes a diagnostic dilemma.

## Case presentation

A three-year three-month-old, previously healthy girl was admitted with complaints of fever for five days, red eyes, and rashes all over her body for three days. The contact history for Koch was negative. She was immunized for her age according to the Indian Academy of Pediatrics vaccination schedule. Positive findings on the initial examination included fever (103 ℉), tachycardia, congested oral mucosa, strawberry tongue and cracking of the lips, maculopapular rashes over the trunk and thighs, a significant right-sided middle cervical lymph node, palmar erythema, and bilateral non-purulent conjunctivitis, which fulfilled the criteria for Kawasaki disease. Supportive laboratory investigations (Table 1) showed anemia for age, raised inflammatory markers (CRP-20.5 mg/dl, ESR-63 mm 1st hour), hypoalbuminemia (serum albumin-2 g/dl), hyponatremia (serum sodium-126 meq/L), and pyuria (8-10 Pus cells) typically suggestive of KD. Echocardiography was within normal limits [z score of left anterior descending coronary artery 1.46, z score of right coronary artery 1.74, with no left ventricular dysfunction, mitral regurgitation, or pericardial effusion]. Hence, she was diagnosed with Kawasaki disease, and IVIG was given at 2 g/kg along with aspirin in the anti-inflammatory dose and other supportive treatments, including ceftriaxone as a first-line empirical antibiotic. She became afebrile 24 hours after completing the IVIG infusion, but her fever reappeared after two days.

**Table 1:** Laboratory data Hb: hemoglobin; HCT: hematocrit; TLC: total leucocyte count; CRP: C-reactive protein; ESR: erythrocyte sedimentation rate; ALT: alanine transaminases; AST: aspartate transaminases; PT: prothrombin time; APTT: activated partial thromboplastin time; HPF: high power field

Laboratory investigations	Value				Reference range
	5^th^ day of fever	9^th^ day of fever	11^th^ day of fever	14^th^ day of fever	(and unit)
Hb	10.1	9.7	10.6	9.9	11.5–16.5 g/dl
HCT	33.8	30.3	34.3	32.4	35–50%
TLC	7550	13,260	19,310	25,920	4000–11,000 per cu mm
Neutrophil	88	69	77	70	60–70 %
Lymphocyte	9	24	18	23	30–40 %
Platelet count	1.85	3.10	4.05	5.82	1.5–4.5 L/cu mm
CRP	20.5	7.98	10.17	4.20	0.08–0.79 mg/dl
ESR (1^st^ hr)	63	72	48	68	3–12 mm
Serum bilirubin	0.59	-	0.44	-	0.2–1.0 mg/dl
ALT	15.30	-	16.10	-	5–45 U/L
AST	32	-	35	-	0–35 U/L
Urea	72.8	18.9	-	-	11–36 mg/dl
Serum creatinine	0.7	0.71	-	-	0.3–0.7 mg/dl
PT	15.4	-	-	-	10.3–16.5 seconds
INR	1.11	-	-	-	0.8–1.2
APTT	30.5	-	-	-	23.4–38.4 seconds
Dengue antigen	Negative	-	-	-	-
Serum amylase	74.5	-	-	-	60–100 su
Serum sodium	126	132	136	-	138–145 mEq/l
Serum potassium	3.4	3.2	4.6	-	3.5–5.5 mEq/l
Serum chloride	95	102	101	-	95–108 mEq/l
Serum calcium	7.81	-	-	-	8.8–10.8 mg/dL
Serum protein	5	-	7	7.5	6.1–7.9 g/dl
Serum albumin	2	-	2.85	3.17	3.5–5.6 g/dl
Urine pus cells	8–10	2–3	-	-	/HPF
Urine nitrite	Negative	Negative	-	-	-
Serum ferritin	-	-	459.2	-	11–306.8 ng/ml

The blood culture was sterile, but the urine culture showed the growth of Klebsiella pneumoniae. The antibiotic was upgraded to Cefoperazone-Sulbactum as per sensitivity. She persisted in having high-grade fever spikes even after four days of antibiotics. Urine routine and microscopy repeated on day 9 of fever showed 2-3 pus cells/hpf, and urine culture was sterile, but the fever persisted. Repeat investigations on day 14 of fever showed neutrophilic leukocytosis (25920, 70% polymorphs), high CRP (4.2 mg/dl), and high ESR (68 mm first hour). She was diagnosed with IVIG-resistant KD. A second dose of IVIG, along with oral corticosteroids and aspirin, was given. She remained afebrile after the second dose of IVIG by day 16 of fever onset and was discharged by day 19.

The child has been on weekly follow-up for the past eight weeks. Serial echocardiography has been within normal limits. Laboratory parameters improved after two weeks of hospital discharge (hemoglobin 12.2 g/dl, leucocyte count 10490/cu mm, neutrophil 47%, lymphocyte 42%, platelet count 4.18 L/cu mm, CRP 0.14 mg/dl, and ESR 43 mm in the first hour). Aspirin was stopped six weeks after discharge, and she remains asymptomatic to this date.

## Discussion

The diagnosis of Kawasaki disease requires the presence of a fever lasting at least five days without any other explanation combined with at least four of the five criteria, which include bilateral bulbar conjunctival injection, oral mucous membrane changes, peripheral extremity changes, polymorphous rash, and cervical lymphadenopathy [3].

Diagnosis of incomplete KD is made when at least five days of fever are accompanied by two or three of the five clinical criteria as stated above, along with a positive echocardiogram, a high CRP (>3 mg/dl), an ESR (>40 mm first hr), or three or more laboratory findings [3]. Positive laboratory findings include anemia for age, thrombocytosis (>4,50,000) after the seventh day of fever, albumin <3 g/dl, elevated ALT levels, TLC >15,000/cu mm, and urine ≥ 10 pus cells/HPF. A positive echocardiogram means any of three, including the z score of the left anterior descending coronary artery (LADCA) ≥ 2.5; the right coronary artery (RCA) ≥2.5/coronary artery aneurysm; or ≥3 out of decreased left ventricular function, mitral regurgitation, pericardial effusion, or z scores in LADCA or RCA 2-2.5.

IVIg-resistant Kawasaki disease is defined as persistent or recrudescent fever 36 hours after the initial IVIg infusion [3]. IVIG-resistant KD cases are at a higher risk of developing coronary artery aneurysms and, hence, need additional therapy to control inflammation [9]. Treatment options for such cases include a second dose of IVIg alone, IVIg with prednisolone, infliximab, cyclosporine, anakinra, cyclophosphamide, and plasma exchange [3].

Benseler et al., in a single-center retrospective study of 129 typical KD patients, reported an incidence of concurrent infections of 33% [10]. There are few case reports of Kawasaki disease with urinary tract infection (UTI) in children. Husain and Al-Rashid have reported KD in association with UTI in a two-month-old infant [11]. Similarly, Jan et al., in their retrospective cohort study, also found that 10.7% of all KD cases with pyuria were associated with a culture-proven UTI [12]. Natarajan and Rothman have reported IVIg-resistant KD with UTI, although such cases are scarce in the literature [13].

## Conclusions

Co-infection with IVIg-resistant Kawasaki disease is a rare entity. Complications are more common in IVIg-resistant KD cases than in classical KD cases. For successful treatment of co-infection, there should be a high index of suspicion for such cases with fever persisting despite treatment with IVIg and antibiotics.

Concerned subspecialties like pediatric cardiologists, pediatric intensivists, infectious disease specialists, and pediatric rheumatologists should be involved in decision-making in such cases. Delays in diagnosing and treating appropriately may lead to complications like coronary artery aneurysm/thrombosis, myocardial infarction leading to myocardial dysfunction, valvular regurgitation, arrhythmias, and even death.
